# Numerical computation of 3D Brownian motion of thin film nanofluid flow of convective heat transfer over a stretchable rotating surface

**DOI:** 10.1038/s41598-022-06622-9

**Published:** 2022-02-17

**Authors:** Zeeshan, Haroon Ur Rasheed, Waris Khan, Ilyas Khan, Nawa Alshammari, Nawaf Hamadneh

**Affiliations:** 1 Department of Mathematics and Statistics, Bacha Khan University Charsadda, Charsadda, 25000 KP Pakistan; 2 Abdul Wali Khan University Mardan, Mardan, 25000 KP Pakistan; 3 Department of Mathematics and Statistics, Hazara University Mansehra, Dhodial, KP Pakistan; 4 Department of Mathematics, College of Science Al-Zulfi, Majmaah University, Al-Majmaah, 11952 Saudi Arabia; 5 Department of Basic Sciences, College of Science and Theoretical Studies, Saudi Electronic University, Riyadh, 11673 Saudi Arabia

**Keywords:** Mathematics and computing, Physics

## Abstract

This research examines the thin-film nanomaterial movement in three dimensions over a stretchable rotating inclined surface. Similarity variables are used to transform fundamental systems of equations into a set of first-order differential equations. The Runge–Kutta Fourth Order approach is utilized for numerical computations. The impact of embedded parameters (variable thickness, unsteadiness, Prandtl number, Schmidt number, Brownian-motion, and thermophoretic) is examined carefully. Physically and statistically, the indispensable terms namely Nusselt and Sherwood numbers are also investigated. Results indicated that, as the dimensionless parameter S raises, the temperature field decreases. In reality, as the values of S increases, heat transmission rate from the disc to the flowing fluid reduces. Internal collisions of liquid particles are physically hampered at a low rate. The momentum boundary layer is cooled when the parameter S is increased, as a consequence local Nusselt number rises. Sherwood number decreases as the parameter S increases because of inter collision of the microscopic fluid particles. Enhancing in the apparent viscosity and concentrations of the chemical reactions, a higher Schmidt number, Sc, lowers the Sherwood number. With increasing values of Prandtl number the Nusselt number decreases. For validation purpose, the RK4 method is also compared with homotopy analysis method (HAM). The results are further verified by establishing an excellent agreement with published data.

## Introduction

In the sphere of chemistry and applied sciences, the development of liquid condensate from a cool, saturated vapor is crucial. Many researchers have looked into this phenomenon under a variety of circumstances. Gregg et al.^1^ used the centrifugal force characteristic on a cold spinning disc to investigate the removal of moisture. They converted the fundamental flow equations into highly nonlinear equations and attempted the numerical solution for liquid layer thicknesses of finite and varying thickness. Hudson et al.^2^ carried their work a step further by incorporating vapour drag. The theory of Sarma et al.^3^ has been expanded to include the adhesion term at the plate surface.

The mutual fluid, which has a poor thermal conductivity, is employed as a basis fluid in much of the available literature. The outputs of these types of heat systems are extremely low. Nanoparticles are tiny particles that are injected inside a base fluid to improve the chemical property of consideration fluid. Hatami^4^ investigated the discharge of a nanomaterial across a revolving, inclined plane. Significant physical results for cooling purposes were preserved.

The application of time-dependent flow field in engineering and physical science is equally significant. In situation of porous medium, Attia^5^ examined the flow behavior around a circular cylinder. The flow rate through a porous medium was examined by Bachok et al.^6^. They employed nanofluids to heat transfer. The numerical findings of unsteady magnetohydrodynamic streaming of a flow of nanofluid across a permeable upwardly expanding medium were observed by Freidoonimehr et al.^7^. Makinde et al.^8^ observed the impact of changing viscosity on nanoliquid streaming. Akbar et al.^9^ used a magnetism to analyze a two-dimensional stream of a nanomaterial and used shooting approach to find the numerical solution. During partial slip scenarios, Chung et al.^10^ analyzed micro-polarnanomaterial moment generated by rotating disc. We can look at the most recent works^11–13^ for a thorough analysis as well as in investigation of magnetohydrodynamicnanofluid streaming with various properties. The particles employed in Nano fluids are often made up of atoms (Al, Cu) oxides (Al2O3), nitrides (AlN, SiN), or thermoplastic elements (Polymer, Graphene oxide), with a propagation liquid such as water as well as ethylene glycol serving as the conventional fluids. As a base lubricant, oily chemicals, biofluids, and polymer coatings can be employed. Nanomaterials have sizes between 1 and 100 nm. Nano fluids typically include up to 5% aspect ratio of nanomaterials to confirm greater heat-transfer improvements. Nano fluids have peculiar properties that allow them to be used in a variety of applications involving hypervisor engines, pharmaceutical procedures, fuel cells, integrated circuits, as well as heat transmission. These have been extensively utilized in engineering-applications for ship soil in boilers exhaust gases heat dissipation and defense, as well as in space flight, grinding, nuclear plant and pressurizer, chiller, residential refrigerator, and engines vehicle plasma management. Nano fluids greatly improve the convection of the polymer matrix; hence researchers are particularly interested in studying the flow with nanofluids. Nanoparticles are also exceedingly consistent, with no additional problems like pressure decrease, erosion, or sedimentation. Choi^14^ was the first to develop nanofluids technology. Thermodynamic management is a challenge in ferromagnetic materials and ultrafast computing applications. Rheological characteristics of nanofluids have been a focus of interest and research numerous applications in electronic freezing and heat transmission. Xie et al.^15^ measured the heat capacity as well as viscous of ethylene polyvinyl nanofluids to investigate their thermal transport capabilities. Xie et al.^15^ explored the rheological impacts as well as transmission of heat characteristics of Al2O3 Nanofluids focusing on 45% ethylene glycol and 55% water in another investigation. Carbon-based nanostructures have gained popularity since the development of nanotubes (CNTs) in 1991 because to their distinctive facial, radioactive, physical, and electromagnetic capabilities. Yu et al.^16^ used a green technique to manufacture silver nanoparticle-decorated intra nanofluids (MWNT) blends (Ag-NPs). A single layer of graphite, of 2D form of carbon, has been discovered to exhibit good crystal quality and efficient electrical transport at ambient temperature in the event of graphene. According to Xie and Chen^17^, it has evolved into a remarkable material with unusual physical, biological, and structural features. Yu et al.^18^ found that Nano fluid including grapheme oxide nanoparticles have much greater thermodynamic properties than the base fluid. Because of the various potential applications, it is critical to learn further about heat exchange characteristics of water. Among the most contentious issues in hybrid nanofluid is efficient thermal diffusivity. Furthermore, due to the complexity and variability, physical nature is still poorly understood. Brownian motion generated convection and efficient transmission through propagating nanoparticle routes are indeed the most two common mechanisms for increased heat transmission in nanofluids, according to a rigorous investigation. While its effects of various parameters like nanotube concentration and aggregate intensity have been studied in literatures^19–22^, yet an entire mechanistic explanation is not provided. The exceedingly intricate processes of heat transmission and the interplay involving thermal conductivity, pore size with nanoparticle concentration make it difficult to evaluate the actual thermophysical properties analytically. As a result, the precise mechanism of convection in nanofluids is currently unknown. To close this gap, Ellahi^23^ used the customs and beliefs to establish analytical formulas for optimal nanoparticle concentration while accountingheat convection due to Brownian moment of nanomaterials. Analogously, Mustafa et al.^24^ focused into the progress of nanoparticle survey by incorporating fractal theory as well as conveying a nonlinear model based on the fractal dispersion and heat condensation for nanomaterial fluid owing to Brownian moment of nanomateriales. A thorough explanation of the Nano fluid was addressed by Akbar and Nadeem^25^. Nowar^26^ investigated Maxwell fluid’s effect and variable viscosity for non-Newtonian flow of nanofluid in a pipe. Choi et al.^27^ described how a nanofluid passes over a stretched surface. Terekhov et al.^28^ investigated wall behavior impact at peristaltic moment of a ferrofluid. Endoscopic examination of peristaltic nanomaterial moment was addressed via Yu et al.^29^. In domain of Hall current with permeable medium, Hojjat^30^ addressed nanofluid model for peristaltic flow. The sources^31–39^ contain a comprehensive investigation on various aspects of nanofluids.

An electromagnetic nanofluid is a unique substance that combines the properties of a fluid as well as a magnetic material. Such fluids are used in a variety of applications, including magneto optical wavelength filters as well as other optical materials such as complicated structures and tunable fiber filters, glass panels, and switches. Changing the amplitude of a magnetic field can modify a variety of physical features of all such fluids. Nanofluids based on magnetism are currently being used in a variety of fields, including, biomedicine, pharmacy, and submarine float isolation. In most of biomedical applications which involves Nanofluids, such as drug delivery, magnetic detection, and significant decline in neuroimaging. Because of its application in power sources, MHD accelerator, refrigerated coils, transmission system, electro transformers, and heaters, MHD processes are important. Because of the richness of this concept, various scholars have focused on MHD motions. Transfer of energy is facilitated by composition gradients, while mass movement is facilitated by a thermal gradient. These characteristics of MHD motion are used in fluid mechanics for suspension and fluid pump, liquids actuators, and transpiration techniques, as well as aerodynamics. Heat transmission in boundary layer flow via stretched surfaces has several applications in the injection molding. The MHD motions inside an electrical conductor liquid, which may manage the impact of cooling, effectively accomplish the quality level of a manufacturing process. Several technological operations, such as glass fiber production, foodstuff and paper production, glass blowing, metal spinning, and metallurgical procedures including crystal production, polyester & rubber sheet preparation, bronze threads enameling and decorating, and many more are significant manufacturing implementations of the problem of viscoelastic mobility and heat transmission beyond a stretched surface. During the production of such sheets, the problems in molten state are stretched from either a gap to reach the appropriate size. The finished product mostly with needed qualities is manufactured due to temperature stretching rate throughout the procedure as well as the stretching cycle. Abu^40^ investigated Brownian motion and thermal flexibility impacts on MHD viscoelastic moment of a nanomaterial via a stretchable porous material. Alim et al.^41^ examine the MHD time dependent motion of a nanoliquid through a longitudinal stretch sheet under suction/injection.There's been enough investigation on displacement past stretched surfaces. Khan^42^ was the first one to address flow on smooth and substantial continuous surfaces. Gul et al.^43^ looked at the fluid flow an extendable barrier by assuming that only the surface velocity varied linearly from the slit. MHD solution of a viscoelastic-non-Newtonian liquidover a stretching disc was discovered by Gul et al.^44^. Aziz et al.^45^ studied the impact of varied Al2O3 water Nano liquid characteristics on the enhancement of heat transmission in entropy generation. Khan et al.^46^ studied heat generation using a flat—plate absorber and a nanofluids with changeable characteristics. In study, thin fluids film fluxes are receiving a lot of attention. Variable parameters of a thin fluid flow through an extending region were studied by Qasim et al.^47^. Prashant et al.^48^ intensively reviewed the flow as well as heat transmission non-Newtonian liquid via a porous media through shrinking surface. Gireesha et al.^49^ explored thermophoresis and nonlinear thermal MHD thin film fluid of second grade with temperature dependent viscosity through a stretched sheet, as well as heat and mass transport. Time-dependent stretched surface with heat generation, Wang^50^ observed thin film flow and heat source. Nazar et al.^51^ investigated Williamson nanomaterial fluid with changing concentration and temperature on the time-dependent stretched. Adopting model of Buongiorno, Emam et al.^52^ investigated the transmission of pressure drop and heat in nanofluids through a time-dependent stretch sheet. Emam et al.^53^ demonstrated heat transfer over magnetic field using time-dependent stretch sheet along thermal radiation of thin flow. The flow phenomena and heat transmission past small cylinders has undergone a significant revolution in recent years. The unavoidable needs for slim devices that reduce drag while delivering whole lift in order to keep the body afloat in specific scenarios. In narrow cylinder, radius can be like the boundary layer thickness having is axisymmetric behavior rather than two-dimensional, and the governing equation includes a transverse curve that impacts the temperature and velocity fields through force.

A normal curvature has an effect on coefficient of skin friction and rate of heat transfer at the wall, which is relevant to this concept. The preparation of combustion chambers, chimney stacks, coolers, offshore structures, thin film deposition, and paper manufacture are all examples of flow past a cylinder and related transfer of heat properties. Sheikoleslami et al.^54^ was the one to evaluate the third-grade non-linear viscous fluid flow via a stretched circular tube. Ahmad et al.^55^ concentrate the fluid motion beside a extending tube utilizing Keller-box approach for solution. The analogous results of the natural convection investigation over a quasi-stretched cylinder were obtained by Sheikholeslami^56^. Wang^57^ provided a computational solution of MHD Newtonian fluid moment through a stretched disc. Nanomaterial mobility with heat as well as attractive field over extending surface was described by Kleinstreuer et al.^58^. Koo^59^ looked at how incompressible Newtonian fluid moves and heat transfer across a stretchable cylinder with variable viscosity. Prasher et al.^60^ examined effect of uniform pressure over stretchable cylinder in presence of nanoparticles liquid. Jang^61^ focused at simulated fluid flow including heat transfer within micro systems. Other studies of nanofluids and many intriguing challenges with regard to various features can be found in^62–65^. The effect of the wall temperature on laminar heat transfer in a rotating disk and turbulent heat transfer at constant temperature of density of heat flux are investigated by Shevchuk^66,67^. Shamshuddin and Mabood investigated the thermo solutal micro polar nanofluid with chemical reaction over stretching sheet^68^. Shamshuddin et al.^69,70^ studied the 3D Williamson fluid and nanofluid boundary layer flow through stretching sheet with sanctions and heat generations. Rezwan et al.^71^ and Salawu et al.^72^ studied Ferromagnetic/nonmagnetic nanofluid and Oldroyd-8 constant fluid with thermal ignitions respectively. Beg et al.^73,74^ investigated the experimental study of rheology and lubricity of nanofluid and naopolymer flow with nanoparticles volume fraction effect. Shamshuddin et al.^75–77^ analyzed the radioactive Marangoni convection in Cu-water based nanofluid flow with porous media over a disk. Shevchuk^78^ explained the convective heat/mass transfer in rotating flows in detail.

The goal of this work is to investigate the spraying nanomaterial fluid across an angled rotating disc for cooling purposes, in light of the preceding critical debate. Through suitable transformations, the basic equations of continuity, momentum, thermal boundary layer, as well as mass for time dependent density flow are rehabilitated to non-linear ordinary differential equations (ODEs). To generate first order ODEs, these are additionally distorted in order to obtain numerical solution. The numerical solutions of the transformed first order ODEs were achieved using the RK4 technique. The numerical results are indeed validated using the HAM for the sake of confirmation. Furthermore, we verified the acquired results by establishing a comparing with previous literatures, and we discovered an outstanding match, confirming the accuracy of the current communication.

## Modeling of the problem

**Figure 1:**
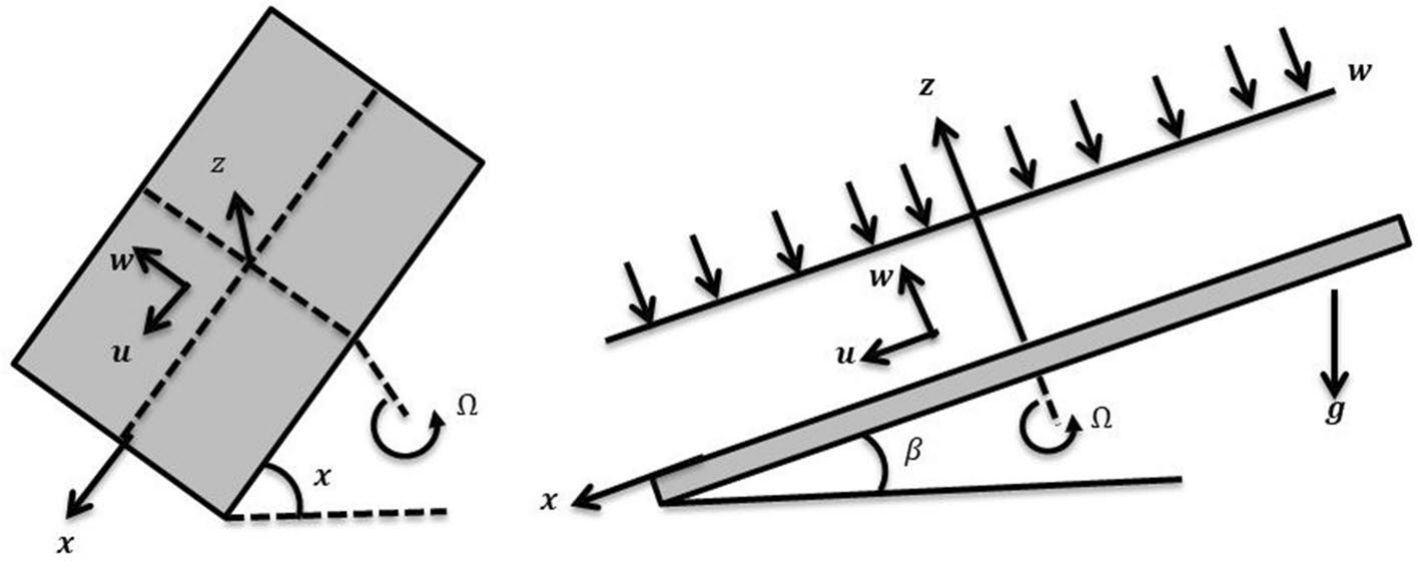
Geometry of the problem.

Take a rotating disc with a 3D unsteady nanomaterial thin-film moment. As seen in Fig. 1, the disc rotates with angle $\Omega$. The horizontal line has been at an inclination $\beta$ with the slanted disc. The nanomaterial sheet thickness is denoted by $h$, as well as the spray speed is indicated by $W$. Because the fluid film's thickness is already so thin in comparison to the radius of the disc, the terminal effect is neglected. The gravitation force $\overline{g}$ is exerting in the negative direction as it often does. The temperature ${\theta }_{0}$ is at the film surface, whereas ${\theta }_{w}$ is over the disc. The Concentration happening on surface film is ${C}_{0}$, while the concentration on surface of is ${C}_{h}$.

The constant pressure ${p}_{0}$ held at surface film, is merely the function of z. Omitting viscous dissipation, and for unsteady flow essential model equations for continuity, momentum boundary layer, temperature boundary layer as well as mass are provided^2–4,6,7,9^

1 $$\frac{\partial {\varvec{u}}}{\partial x}+\frac{\partial {\varvec{v}}}{\partial y}+\frac{\partial {\varvec{w}}}{\partial z}=0,$$

2 $$\rho_{f} \left( {\frac{\partial u}{{\partial t}} + u\frac{\partial u}{{\partial x}} + v\frac{\partial u}{{\partial y}} + w\frac{\partial u}{{\partial z}}} \right) = \mu \left( {\frac{{\partial^{2} u}}{{\partial x^{2} }} + \frac{{\partial^{2} u}}{{\partial y^{2} }} + \frac{{\partial^{2} u}}{{\partial z^{2} }}} \right) + \rho_{f} \overline{g}\sin \beta ,$$

3 $${\rho }_{f}\left(\frac{\partial {\varvec{v}}}{\partial t}+u\frac{\partial {\varvec{v}}}{\partial x}+v\frac{\partial {\varvec{v}}}{\partial y}+w\frac{\partial {\varvec{v}}}{\partial z}\right)=\mu \left(\frac{{\partial }^{2}{\varvec{v}}}{\partial {x}^{2}}+\frac{{\partial }^{2}{\varvec{v}}}{\partial {y}^{2}}+\frac{{\partial }^{2}{\varvec{v}}}{\partial {z}^{2}}\right),$$

4 $$\rho_{f} \left( {\frac{\partial w}{{\partial t}} + u\frac{\partial w}{{\partial x}} + v\frac{\partial w}{{\partial y}} + w\frac{\partial w}{{\partial z}}} \right) = \mu \left( {\frac{{\partial^{2} w}}{{\partial x^{2} }} + \frac{{\partial^{2} w}}{{\partial y^{2} }} + \frac{{\partial^{2} w}}{{\partial z^{2} }}} \right) - \rho_{f} \overline{g}\cos \beta - p_{z} ,$$

5 $$\frac{\partial \theta }{\partial t}+u\left(\frac{\partial \theta }{\partial x}\right)+v\left(\frac{\partial \theta }{\partial y}\right)+w\left(\frac{\partial \theta }{\partial z}\right)=\alpha \left(\frac{{\partial }^{2}\theta }{\partial {x}^{2}}+\frac{{\partial }^{2}\theta }{\partial {y}^{2}}+\frac{{\partial }^{2}\theta }{\partial {z}^{2}}\right) -\frac{{\left(\rho {c}_{p}\right)}_{p}}{{\left(\rho {c}_{p}\right)}_{f}}\left[{D}_{B}\left\{\frac{\partial C}{\partial x}\cdot \frac{\partial \theta }{\partial x}+\frac{\partial C}{\partial y}\cdot \frac{\partial \theta }{\partial y}+\frac{\partial C}{\partial z}\cdot \frac{\partial \theta }{\partial z}\right\}+\frac{{D}_{\theta }}{\theta }\left\{{\left(\frac{\partial \theta }{\partial x}\right)}^{2}+{\left(\frac{\partial \theta }{\partial y}\right)}^{2}+{\left(\frac{\partial \theta }{\partial z}\right)}^{2}\right\}\right],$$

6 $$\frac{\partial C}{\partial t}+u\left(\frac{\partial C}{\partial x}\right)+v\left(\frac{\partial C}{\partial y}\right)+w\left(\frac{\partial C}{\partial z}\right)={D}_{B}\left(\frac{{\partial }^{2}C}{\partial {x}^{2}}+\frac{{\partial }^{2}C}{\partial {y}^{2}}+\frac{{\partial }^{2}C}{\partial {z}^{2}}\right)+\left(\frac{{D}_{\theta }}{{\theta }_{0}}\right)\left(\frac{{\partial }^{2}\theta }{\partial {x}^{2}}+\frac{{\partial }^{2}\theta }{\partial {y}^{2}}+\frac{{\partial }^{2}\theta }{\partial {z}^{2}}\right),$$

With boundary conditions

7 $$\begin{array}{l}u=-\Omega y,v=-\Omega x,w=0,\theta ={\theta }_{w},C={C}_{h},\text{ at }z=0\\ {u}_{z}=0,{v}_{z}=0,w=-W,\theta ={\theta }_{0},C={C}_{0},p={p}_{0},\text{ at }z=h\end{array}.$$

We assume the transformations^4,9,13^

8 $$\begin{array}{ll}& u=\frac{-\Omega y}{1-bt}g(\eta )+\frac{\Omega x}{1-bt}{f}^{\prime}(\eta )+\frac{\overline{g}}{\sqrt{1-bt}}k(\eta ){\rm sin}\frac{\beta }{{\Omega }^{\prime}},\\ & v=\frac{-\Omega x}{1-bt}g(\eta )+\frac{\Omega y}{1-bt}{f}^{\prime}(\eta )+\frac{\overline{g}}{\sqrt{1-bt}}h(\eta ){\rm sin}\frac{\beta }{{\Omega }^{\prime}},\\ & w=-2\sqrt{\frac{\Omega {v}_{f}}{1-bt}}f(\eta ),\eta \theta (\eta )=\frac{\theta -{\theta }_{w}}{{\theta }_{0}-{\theta }_{w}},\eta \phi (\eta )=\frac{C-{C}_{w}}{{C}_{0}-{C}_{w}}\\ & \eta =z\sqrt{\frac{\Omega }{{v}_{f}(1-bt)}}\end{array}.$$

The transformations described in Eq. (8) are then placed into Eqs. (2)- (7), resulting in Eq. (1) being confirmed similarly and Eq. (2)–(6) yielding:

9 $${f}^{\prime\prime\prime}-{\left({f}^{\prime}\right)}^{2}+{g}^{2}-2f{f}^{\prime\prime}-S\left({f}^{\prime}+\frac{\eta }{2}{f}^{\prime\prime}\right)=0,$$

10 $${K}^{\prime\prime}-K{f}^{\prime}-hg+2f{K}^{\prime}+1-\frac{S}{2}\left(K+\eta {K}^{\prime}\right)=0,$$

11 $${g}^{\prime\prime}-2g{f}^{\prime}+2{g}^{\prime}f-S\left(g+\frac{\eta }{2}{g}^{\prime}\right)=0,$$

12 $${h}^{\prime\prime}-Kg-h{f}^{\prime}+2f{h}^{\prime}-\frac{S}{2}\left(h-\eta {h}^{\prime}\right)=0.$$

Equations (5) and (6) become if $\theta$ and $C$ are functions of z

13 $${\theta }^{\prime\prime}+2{\rm Pr}f{\theta }^{\prime}+Nb{\phi }^{\prime}{\theta }^{\prime}+Nt{\left({\theta }^{\prime}\right)}^{2}+\frac{S}{2}\left(\eta {\theta }^{\prime}+{\eta }^{2}{\theta }^{\prime\prime}\right)=0,$$

14 $${\phi }^{\prime\prime}+2Scf{\phi }^{\prime}+\frac{Nt}{Nb}{\theta }^{\prime\prime}+\frac{S}{2}\left(\eta {\phi }^{\prime}+{\eta }^{2}{\phi }^{\prime\prime}\right)=0,$$

15 $$\begin{aligned}&f(0)=0,{f}^{\prime}(0)=0,{f}^{\prime\prime}(\delta )=0,g(0)=0,{g}^{\prime}(\delta )=0,\\ &K(0)=0,{K}^{\prime}(\delta )=0,h(0)=0,{h}^{\prime}(\delta )=0,\\ & \theta (0)=0,\theta (\delta )=1,\phi (0)=0,\phi (\delta )=1.\end{aligned}$$

where

16 $${\rm Pr}=\frac{\mu }{{\rho }_{f}},Sc=\frac{\mu }{{\rho }_{f}{D}_{B}},Nb=\frac{(\rho c{)}_{p}{D}_{B}\left({C}_{h}\right)}{\left\{(\rho c{)}_{f}\right\}},Nt=\frac{(\rho c{)}_{p}{D}_{\theta }\left({\theta }_{H}\right)}{\left\{(\rho c{)}_{f}{\theta }_{c}\right\}},S=\frac{1}{\Omega }.$$

δ is thickness of normalized.

17 $$\delta =\varepsilon \sqrt{\frac{\Omega }{{v}_{f}(1-bt)}}.$$

Equation (17) can be calculated using spraying velocity

18 $$f(\delta )=\frac{w}{2\sqrt{\Omega v}}=\alpha .$$

Integrating Eq. (4), we get the pressure.

For exact solution take $Pr=0$ by utilizing $\theta (\delta )=1$, we get

19 $${\theta }^{\prime}\left(0\right)=\frac{1}{\delta }.$$

The dimensionless Nusselt and Sherwood numbers

20 $$Nu=\frac{{\left(\frac{\partial \theta }{\partial z}\right)}_{w}}{{\theta }_{0}-{\theta }_{w}}=\delta {\theta }^{\prime}\left(0\right).$$

21 $$Sh=\frac{{\left(\frac{\partial C}{\partial z}\right)}_{w}}{{C}_{0}-{C}_{w}}=\delta {\phi }^{\prime}(0).$$

## Numerical solution

Equation (22) is incorporated to (9–14) in order to obtain first ODE

22 $$\begin{array}{l}{y}_{1}=\eta ,{y}_{2}=f,{y}_{3}={f}^{\prime},{y}_{4}={f}^{\prime\prime},{y}_{5}=g,{y}_{6}={g}^{\prime},{y}_{7}=K,{y}_{8}={K}^{\prime},\\ {y}_{9}=h,{y}_{10}={h}^{\prime},{y}_{11}=\theta ,{y}_{12}={\theta }^{\prime},{y}_{13}=\phi ,{y}_{14}={\phi }^{\prime}.\end{array}$$

Utilizing Eq. (22), the nonlinear higher order derivative system (9–14) are transformed to the first-order ODEs system^21–27^ which are given below

23 $${{\rm y}}_{4}^{^{\prime}}={\left({y}_{3}\right)}^{2}-{y}_{5}^{2}-2{y}_{2}{y}_{4}+S\left({y}_{3}+\frac{1}{2}{y}_{1}{y}_{4}\right),$$

24 $${{\rm y}}_{6}^{^{\prime}}=2{y}_{5}{y}_{3}-2{y}_{6}{y}_{2}+S\left({y}_{5}+\frac{1}{2}{y}_{1}{y}_{6}\right),$$

25 $${{\rm y}}_{8}^{^{\prime}}={y}_{7}{y}_{3}-{y}_{9}{y}_{5}-2{y}_{2}{y}_{8}-1+\frac{S}{2}\left({y}_{7}+{y}_{1}{y}_{8}\right),$$

26 $${{\rm y}}_{10}^{^{\prime}}={y}_{7}{y}_{5}+{y}_{9}{y}_{3}-2{y}_{2}{y}_{10}-\frac{S}{2}\left({y}_{9}-{y}_{1}{y}_{10}\right),$$

27 $${{\rm y}}_{12}^{^{\prime}}=-2{\rm Pr}{y}_{2}{y}_{12}-Nb{y}_{14}{y}_{12}-Nt{y}_{12}{y}_{12}-\frac{S}{2}\left({\left({y}_{1}\right)}^{2}{y}_{12}+{y}_{1}{y}_{11}\right),$$

28 $${{\rm y}}_{14}^{^{\prime}}=-2Sc{y}_{2}{y}_{14}-\frac{Nt}{Nt}\left(-2{\rm Pr}{y}_{2}{y}_{12}-Nb{y}_{14}{y}_{12}-Nt{y}_{12}^{2}\right)-\frac{S}{2}\left({\left({y}_{1}\right)}^{2}{y}_{14}+{y}_{1}{y}_{13}\right).$$

The initial conditions for the nanomaterial fluid flow by applying Eq. (22) are

29 $$\begin{array}{cc}{y}_{1}=0,& {y}_{2}=0,{y}_{3}=0,{y}_{4}={u}_{1},{y}_{5}=1,{y}_{6}={u}_{2},{y}_{7}=0,{y}_{8}={u}_{3},{y}_{9}=0,\\ & {y}_{10}={u}_{4},{y}_{11}=0,{y}_{12}={u}_{5},{y}_{13}=0,{y}_{14}={u}_{6}.\end{array}$$

For solution of differential equations, a numerical method RK4 is now used, and for the conformation and validation Homotopy Asymptotic Method (HAM) is also applied. Furthermore, the current work is also matched with published literature and an outstanding agreement is found.

## Result and discussion

**Figure 2:**
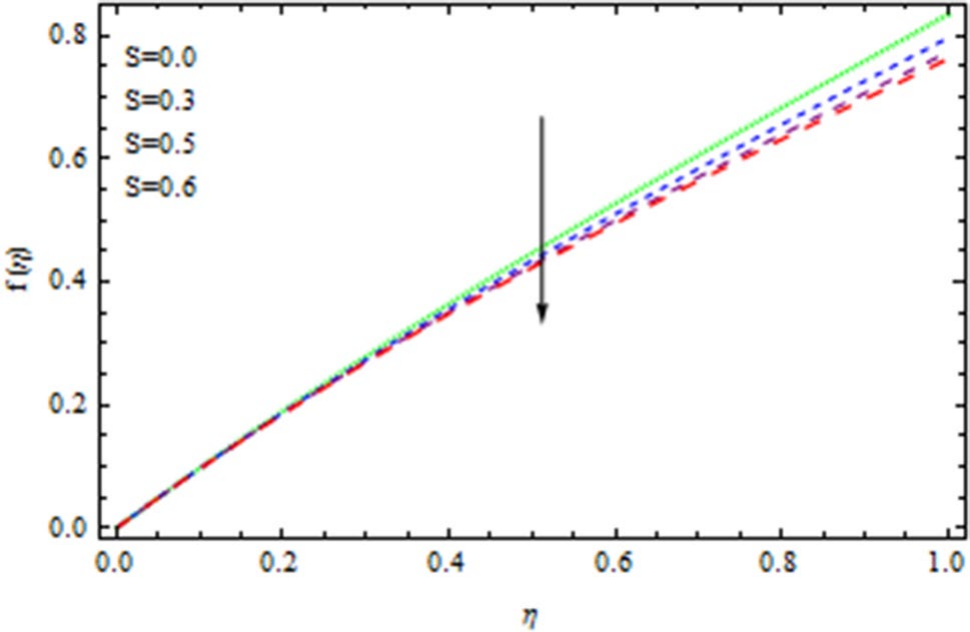
The axial velocity affected by S.

**Figure 3:**
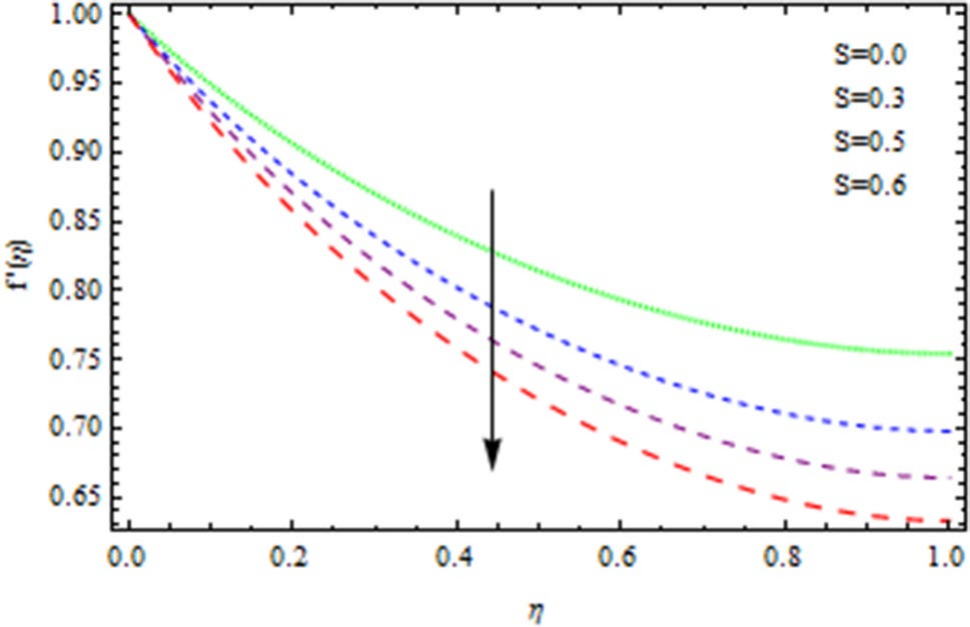
The radial velocity affected by S.

**Figure 4:**
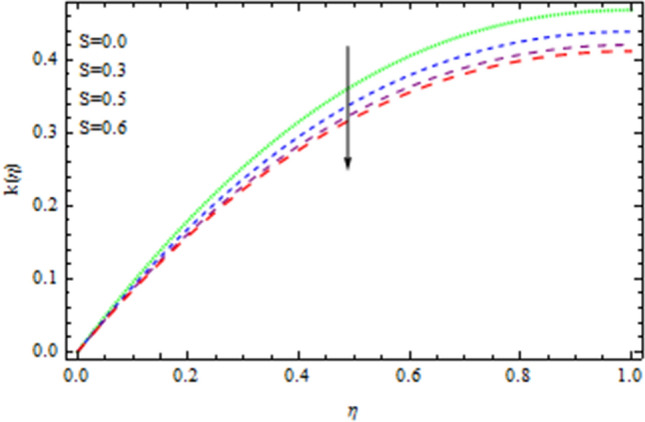
The x-direction draining flow affected by S.

**Figure 5:**
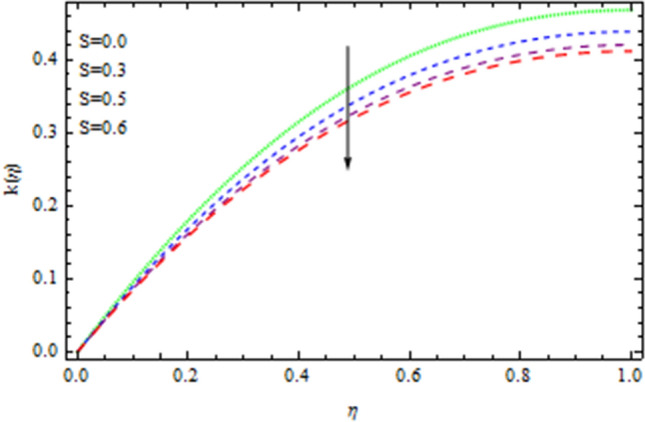
The y-direction induced flow affected by S.

The heat and mass transfer across an unsteady rotational inclined plane using 3D thin-film nanomaterial flow has been investigated. The findings were acquired using the numerical approach Runge–Kutta fourth order method (RK4), while the analytical solution for the validation purposes is obtained using HAM. We used Δr = 0.001 as that of the scale factor and 10–6 and δ = 2, as the resolution threshold during our computation which gives four decimal places accuracy. Figure 1 depicts the current problem physical configuration. A Figures 2, 3, 4, 5 displays the impact of *S* on axial as well as radial velocities, drainage moment, and induced moment, respectively. The variation in fluid moment is depicted by increased quantities of said unsteadiness factor *S*.

**Figure 6:**
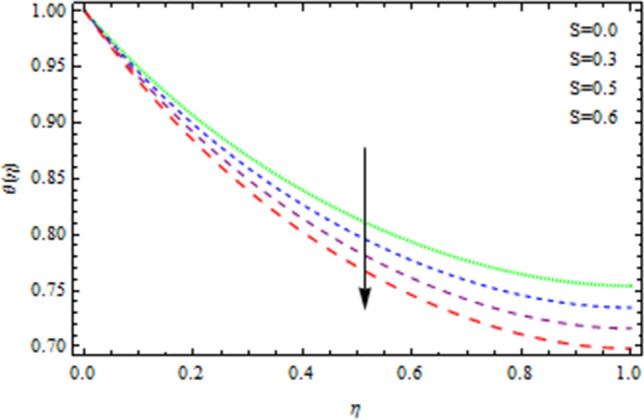
Temperature field affected by S.

**Figure 7:**
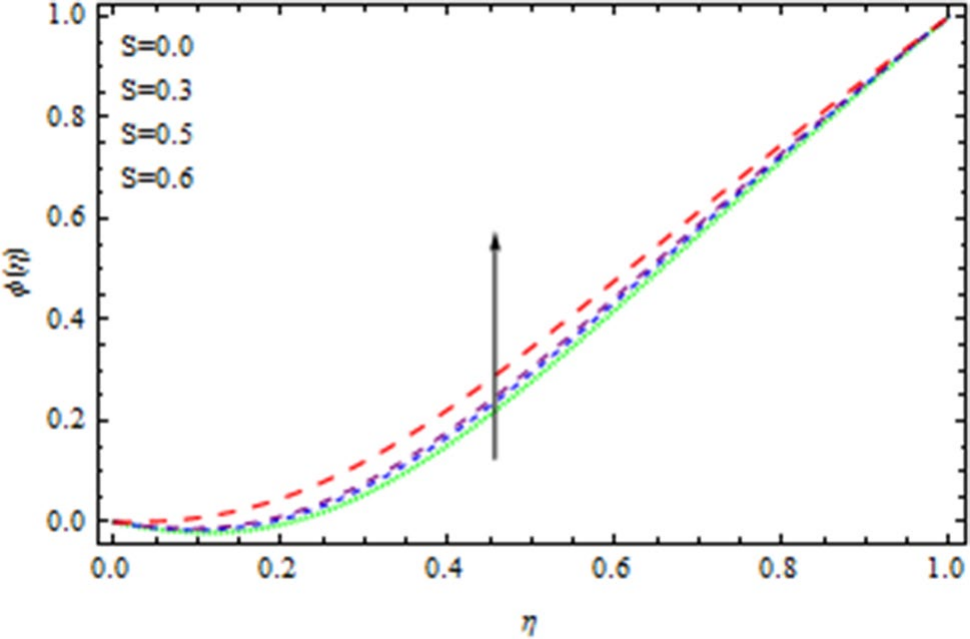
Concentration field affected by S.

For greater values of unsteadiness factor *S*, the momentum thickness grows, and as a consequence, most of the said kinds of fluid flow fall, as seen in the depicted graphs. Figure 6 illustrates that the temperature distribution becomes substantially decreaseswith the increasing values of parameter *S*. In reality, as the values of *S* increases, heat transmission rate from the disc to the flowing fluid reduces. Internal collisions of liquid particles are physically hampered at a low rate. Because as unsteadiness factor *S* is increased, the boundary layer momentum increases, as a consequence, the concentration field also enhances, as seen in Fig. 7.

**Figure 8:**
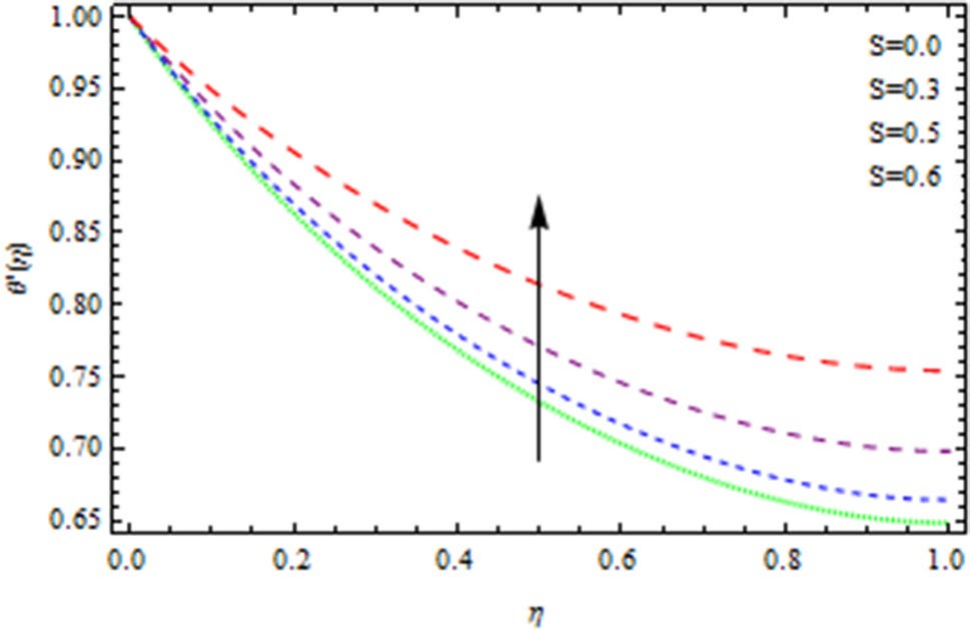
Heat transmission affected by S.

**Figure 9:**
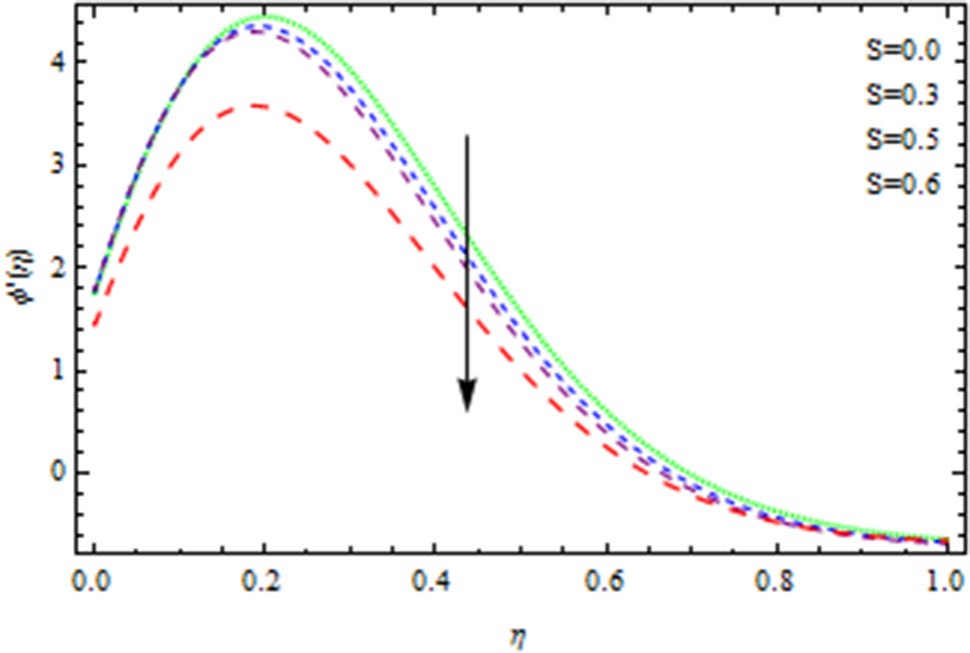
Sherwood number affected by S.

**Figure 10:**
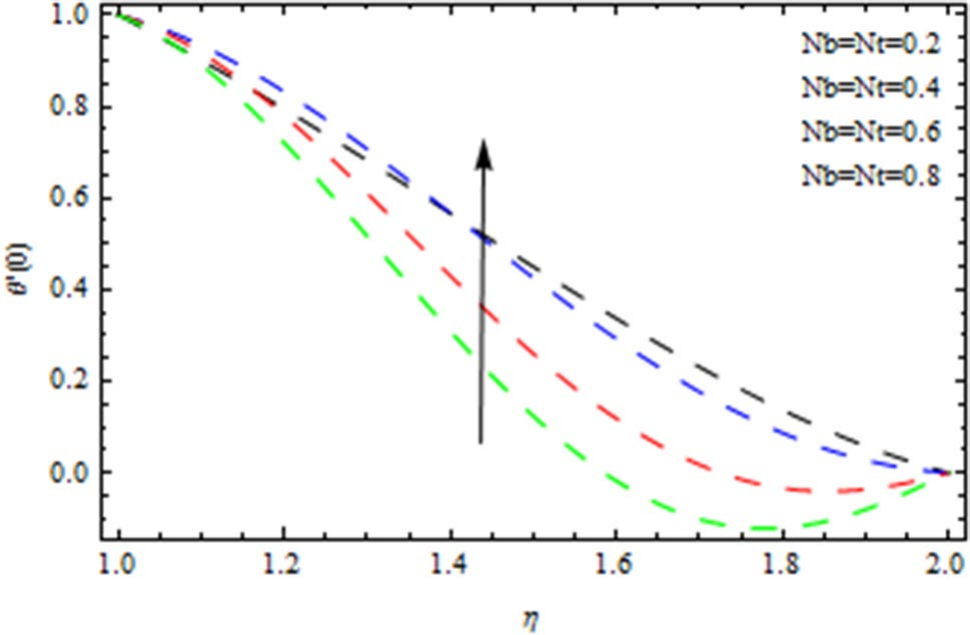
Heat transmission affected by Nt and Nb.

**Figure 11:**
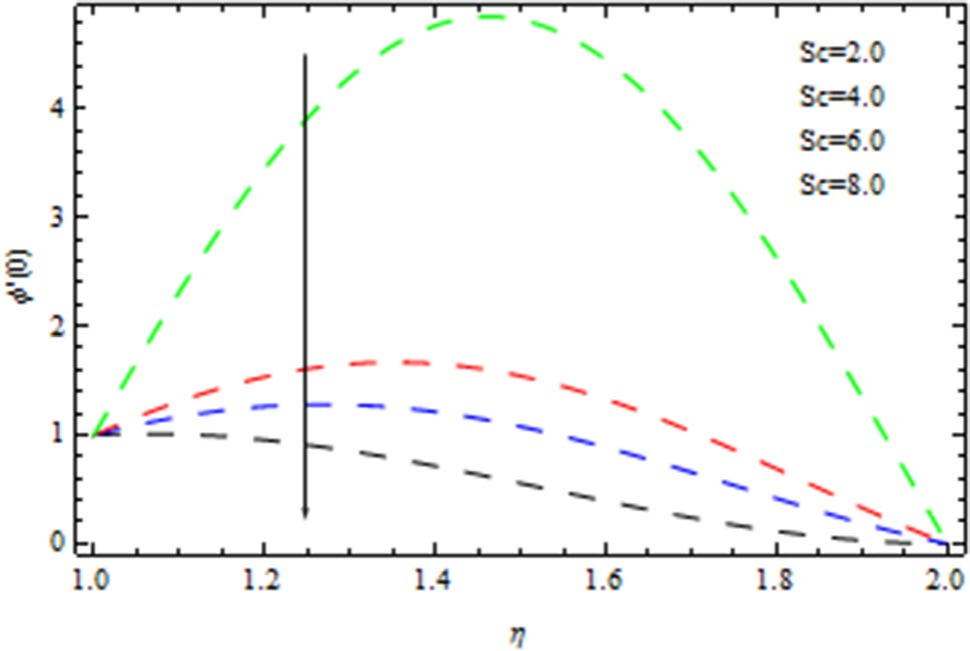
The Sherwood number affected by Sc.

**Figure 12:**
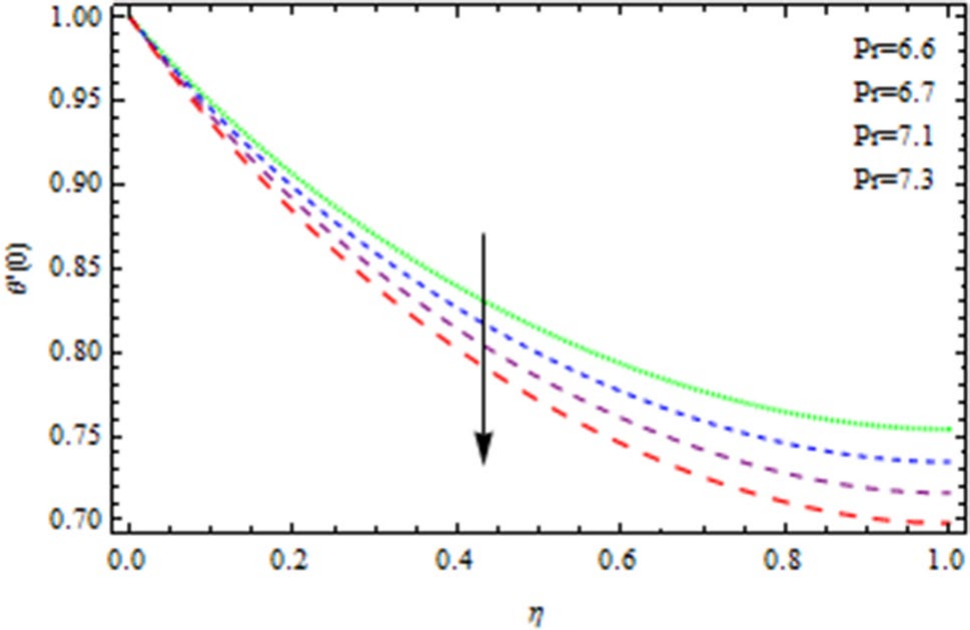
Heat transmission affected by Pr.

The Nusselt number is a non-dimensional number that describes the relation of thermal energy convected towards the liquid to heat energy conducted inside the medium. The Nusselt value is a measurement of heat transfer rate at the barrier that is equivalent to the non-dimensional temperature difference at the surface. Figure 8 depicts the variation of Nusselt number effects by unsteadiness factor *S*. It is clear from Fig. 8 that the momentum boundary layer is cooled when the parameter *S* is increased, as a consequence local Nusselt number rises. The Sherwood number is often used to investigate concentration polarization. The Sherwood number is a non-dimensional number used during mass-transfer operations. It is also known as the mass transfer Nusselt number. In mass-transfer operations, the Sherwood number is a non-dimensional number. It is the proportion of convective mass transfer to diffusive mass transport rate. As illustrated from Fig. 9, that Sherwood number decreases as the parameter *S* increases because of inter collision of the microscopic fluid particles. As depicted in Fig. 10, large amount of *Nt* and *Nb*, enhances heat transfer rate. Nanoparticle movement in nanofluids is caused by thermophoresis and Brownian motion; both have significant influence on the thermo physical properties of nanofluids. The ability of smaller nanostructures to collect at the heated wall and increase the heat transmission rate is demonstrated. Indeed, higher Brownian motion factor *Nb* upsurges the thickness of thermal boundary layer. With increasing Nb, the stochastic collision among nanoparticles and liquid molecules increases, causing a flow to become heated. Figure 11 demonstrates that how concentration rate decreases with changing of Schmidt number Sc. In fact, increasing the $Sc$ parameter enhances kinematic viscosity and increases chemical species concentration, lowering the Sherwood number. The Prandtl number, also known as the Prandtl group, is a non-dimensional number that represents the ratio of momentum to thermal diffusivity. It is a non-dimensional factor equal to $c_{p}$μ/k used in thermal performance computations between a fluid moving and a substantial body, where $c_{p}$ the fluid’s specific heat in unit volume, μ is the kinematic viscosity, and k is its thermal conductivity. Figure 12 depicts the effect of $Pr$ (Prandtl number), on the heat flux. Thermal boundary layer thickness reduces with enhance of $Pr$, and so as a consequence, the cooling rate is decreased.

**Figure 13:**
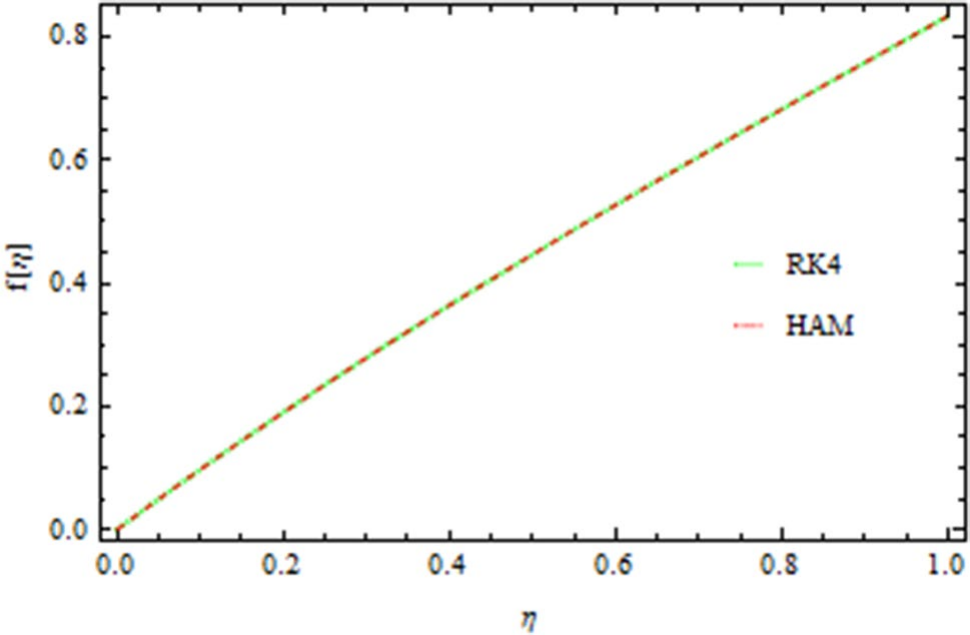
Comparison of RK4 and HAM on the axial velocity.

**Figure 14:**
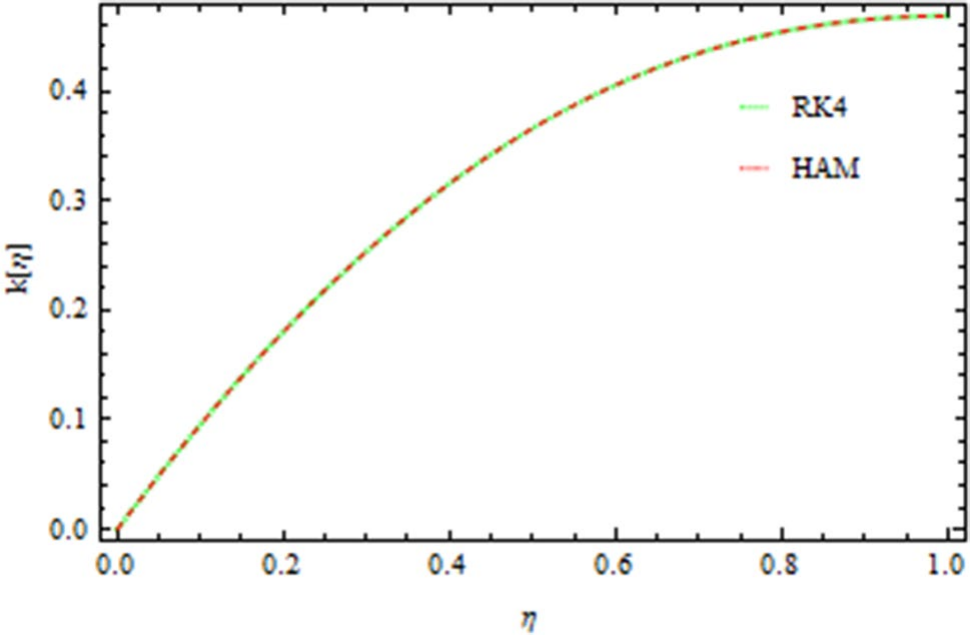
Comparison of RK4 and HAM on the drainage flow.

**Figure 15:**
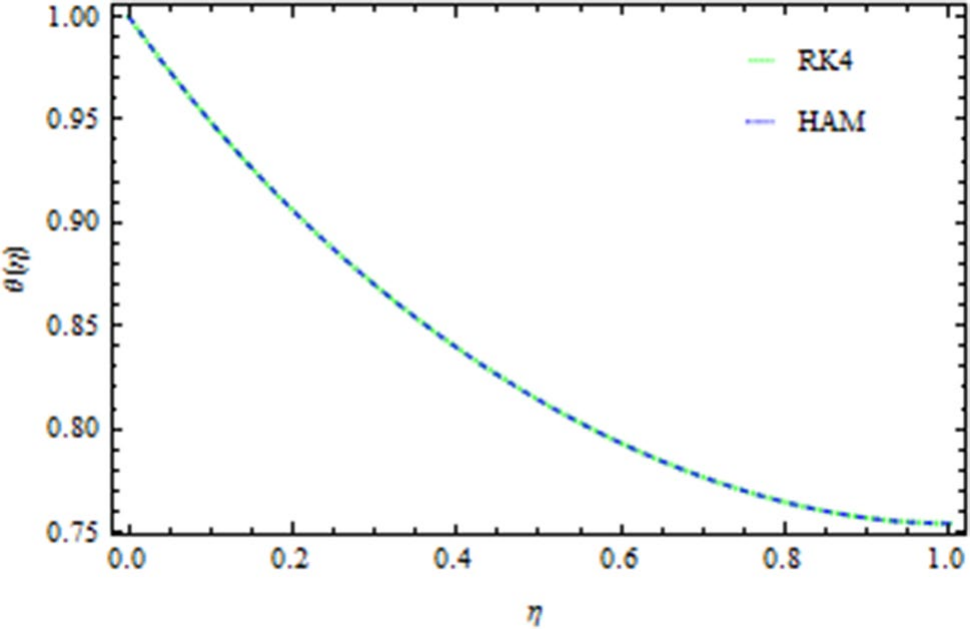
Temperature comparison of RK4 and HAM.

**Figure 16:**
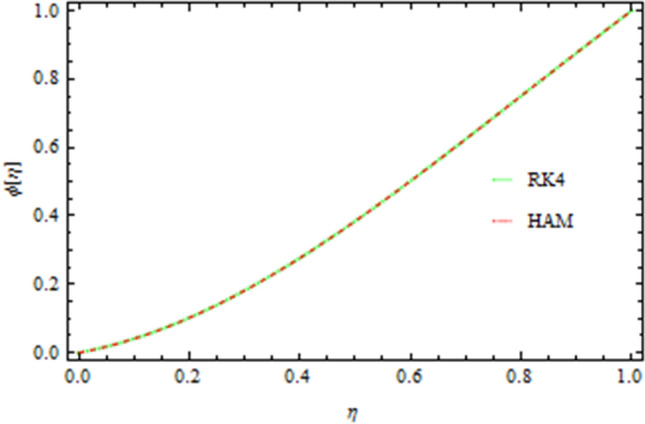
Concentration flow comparison of RK4 and HAM.

The graphical comparison of the RK4 and HAM methods are sketched in Figs. 13, 14, 15 and 16 for the axial and drainage velocities, temperature and concentration fields, respectively, an excellent agreement is noted. Furthermore, the numerical results of the RK4 and HAM methods for the Nusselt number and Sherwood number are given in Table 1. A comparison of the present results with published data is made in limiting sense (see Table 2) which confirms the accuracy and the fact that these results are more general form those in published literature. The Newtonian fluid of the present work can also be obtained by taking $S=0.2,\,Pr=6.2,Nt=Nb=Sc=0{\rm and b}\to 0$ as shown in Table 3.

**Table 1:** Comparison of the present work with published work reported by Gul et al.^13^ with respect to the heat transfer rate and concentration rate, when $Pr=6,Nt=Nb=Sc=S=0.7.S=0.4$.

$\eta$	RK4 $-{\theta }^{\prime}(0)$	HAM $-{\theta }^{\prime}(0)$	RK4 $-{\phi }^{\prime}(0)$	HAM $-{\phi }^{\prime}(0)$
0.1	0.9048	0.9046	1.0947	1.0921
0.2	0.8180	0.8180	1.1790	1.1790
0.3	0.7399	0.7398	1.2509	1.2502
0.4	0.6711	0.6713	1.3081	1.3082
0.5	0.6127	0.6127	1.3480	1.3480
0.6	0.5661	0.5661	1.3683	1.3682
0.7	0.5330	0.5330	1.3662	1.3661
0.8	0.5161	0.5161	1.3386	1.3385
0.9	0.5198	0.5197	1.2805	1.2812
1.0	0.5522	0.5501	1.1831	1.1825

**Table 2:** Comparison of current work with available work reported by Sheikholeslami et al.^4^ for $-{\theta }^{\prime}(0)$ and $-{\phi }^{\prime}\left(0\right)$ fixing $Pr=6.5,Nt=Nb=Sc=S=0.9.S=0.6$.

*η*	RK4 $-{\theta }^{\prime}(0)$	Ref.^4^ $-{\theta }^{\prime}(0)$	RK4 $-{\phi }^{\prime}(0)$	Ref.^4^ $-{\phi }^{\prime}(0)$
0.1	0.9137	0.9046	1.0936	1.0921
0.2	0.8271	0.8180	1.1791	1.1790
0.3	0.7261	0.7398	1.2510	1.2502
0.4	0.6620	0.6713	1.3084	1.3082
0.5	0.6115	0.6127	1.3471	1.3480
0.6	0.5741	0.5661	1.3678	1.3682
0.7	0.5229	0.5330	1.3660	1.3661
0.8	0.5006	0.5161	1.3375	1.3384
0.9	0.5011	0.5197	1.2806	1.2812
1.0	0.5503	0.5501	1.1827	1.1825

**Table 3:** Comparison of Newtonian and non-Newtonian fluid by taking $S=0.2,\,Pr=6.2,Nt=Nb=Sc=0{\rm and b}\to 0.$.

$\eta$	Newtonian	Non-Newtonian	Absolute error
1	1	1	0
1.1	0.03161061	0.03161072	0.125 × 10^−8^
1.2	0.02114131	0.02114143	0.140 × 10^−8^
1.3	0.00510280	0.00510292	0.751 × 10^−9^
1.4	0.01151631	0.01151646	0.213 × 10^−8^
1.5	0.010331134	0.010331135	0.429 × 10^−8^
1.6	0.001411428	0.001411419	0.013 × 10^−9^
1.7	0.006103872	0.006103874	0.025 × 10^−10^
1.8	0.0042015210	0.0042015220	0.224 × 10^−10^
1.9	0.000021152	0.000021164	0.0030 × 10^−10^
2.0	0.00204 × 10^−20^	0.00204 × 10^−22^	0.002 × 10^−25^

## Conclusion

The existing literature focuses primarily on two-dimensional flow problems. The pouring of 3D nanomaterial’s across a stretchable inclined rotatable frame is investigated in this paper. The following is a summary of the new findings in the Numerical and analytical solutions:

- As the dimensionless parameter S raises, the temperature field decreases.In reality, as the values of *S* increases, heat transmission rate from the disc to the flowing fluid reduces. Internal collisions of liquid particles are physically hampered at a low rate.
- The momentum boundary layer is cooled when the parameter *S* is increased, as a consequence local Nusselt number rises.
- Sherwood number decreases as the parameter *S* increases because of inter collision of the microscopic fluid particles.
- Enhancing in the apparent viscosity and concentrations of the chemical reactions, a higher Schmidt umber, Sc, lowers the Sherwood umber.
- With increasing values of Prandtl number the Nusselt number decreases.
